# Differential DNA Methylation Regions in Adult Human Sperm following Adolescent Chemotherapy: Potential for Epigenetic Inheritance

**DOI:** 10.1371/journal.pone.0170085

**Published:** 2017-02-01

**Authors:** Margarett Shnorhavorian, Stephen M. Schwartz, Barbara Stansfeld, Ingrid Sadler-Riggleman, Daniel Beck, Michael K. Skinner

**Affiliations:** 1 Seattle Children's Research Institute, Seattle, Washington, United States of America; 2 Fred Hutchinson Cancer Research Center, Seattle, Washington, United States of America; 3 Center for Reproductive Biology, School of Biological Sciences, Washington State University, Pullman, Washington, United States of America; University of Bonn, Institute of Experimental Hematology and Transfusion Medicine, GERMANY

## Abstract

**Background:**

The potential that adolescent chemotherapy can impact the epigenetic programming of the germ line to influence later life adult fertility and promote epigenetic inheritance was investigated. Previous studies have demonstrated a number of environmental exposures such as abnormal nutrition and toxicants can promote sperm epigenetic changes that impact offspring.

**Methods:**

Adult males approximately ten years after pubertal exposure to chemotherapy were compared to adult males with no previous exposure. Sperm were collected to examine differential DNA methylation regions (DMRs) between the exposed and control populations. Gene associations and correlations to genetic mutations (copy number variation) were also investigated.

**Methods and Findings:**

A signature of statistically significant DMRs was identified in the chemotherapy exposed male sperm. The DMRs, termed epimutations, were found in CpG desert regions of primarily 1 kilobase size. Observations indicate adolescent chemotherapy exposure can promote epigenetic alterations that persist in later life.

**Conclusions:**

This is the first observation in humans that an early life chemical exposure can permanently reprogram the spermatogenic stem cell epigenome. The germline (i.e., sperm) epimutations identified suggest chemotherapy has the potential to promote epigenetic inheritance to the next generation.

## Introduction

Advances in chemotherapy-based curative therapy for childhood cancer have led to a significant improvement in outcome, such that long-term survival approaches 80% [1]. This has resulted in an increasing focus on the later life effects of chemotherapy and quality of life in the growing population of survivors of childhood, adolescent and young adult (AYA) cancer. The toxic effect of cancer chemotherapy on reproductive health is one of the most important challenges faced by male childhood and AYA cancer survivors and is a leading cause of decreased quality of life in this population [2–5]. Osteosarcoma is one of the most common cancers in this population treated with agents such as cisplatin and ifosfamide. This population provides a useful model to investigate potential chemotherapy induced effects on later life reproductive health. Previous studies have demonstrated transient early life toxicant exposures can influence later life health effects and alter epigenetic reprogramming of the germline (i.e. sperm) in animal models [6–8].

Epigenetics is defined as “molecular factors or processes around DNA that regulate genome activity independent of DNA sequence and are mitotically stable” [6, 9]. The currently known epigenetic mechanisms include DNA methylation, histone modifications, selected non-coding RNA and chromatin structure [6]. Although the vast majority of environmental factors can not alter DNA sequence, most have the ability to alter epigenetic programming during development [6, 9]. Early developmental exposures have been shown to alter the epigenetic programming of cells associated with a number of adult onset diseases [6, 9–11]. Environmentally-induced DNA methylation changes in Sertoli or granulosa cells have been shown to associate with testis and ovarian disease in the adult [12, 13]. Environmental epigenetics provides a molecular mechanism for the developmental origins of disease [9]. In the event the altered epigenetic programming occurs in the germline (sperm or egg), the altered epigenetics (e.g. epimutations) have the potential to be transmitted between generations [6–8, 14]. A number of studies have demonstrated that environmental factors (e.g. toxicants and nutrients) following fetal exposure can alter the germline epigenome (e.g. DNA methylation) to then transmit epimutations to subsequent generations [8, 14]. When the germline transmission of epigenetic information occurs between multiple generations in the absence of continuous exposure this is considered to be environmentally-induced epigenetic transgenerational inheritance [6, 7]. This form of non-genetic inheritance is due to the germline transmission of epigenetic information. A number of studies have shown that numerous environmental toxicants such as fungicides [7], plastics [15], pesticides [7] and hydrocarbons [16] can promote the epigenetic transgenerational inheritance of disease [6]. The transgenerational disease observed includes testis, ovary, prostate, mammary, kidney and brain disease [17, 18]. The majority of these transgenerational studies have observed correlations between the phenotypes and differential DNA methylation alterations in the sperm [14]. Therefore, early life environmental exposures can influence the epigenetic programming of the sperm and have the ability to promote epigenetic inheritance to subsequent generations.

Although a previous study in mice focused on genetics [19] demonstrated the ability of the chemotherapy doxorubicin to promote transgenerational disease, the generational impact of chemotherapy in humans has not been thoroughly investigated. Therefore, the current study was designed to investigate the actions of chemotherapy on pubertal males that potentially promote an alteration in epigenetic programming that will result in adult male sperm having epimutations. This requires the spermatogonial stem cell population in the testis to be affected permanently to produce later life effects on the sperm epigenome. Previous studies have demonstrated altered DNA methylation profiles in control versus infertile human male sperm [20] and in rodent sperm following chemotherapy [21]. The presence of sperm epimutations due to adolescent chemotherapy also would suggest for the first time the potential for epigenetic inheritance to the next generation.

## Methods

### Study Population and Samples

The patients were 19–30 year-old male survivors of osteosarcoma recruited between 2/6/2010 and 11/15/2012 from the Seattle Children’s Hospital in Seattle WA and four collaborating institutions (Children’s Hospital of Pennsylvania, Philadelphia, PA; Miller Children’s, Long Beach, CA; Children’s Hospital, University of Minnesota, Minneapolis, MN; and Children’s Hospital, Vanderbilt University, Nashville, TN). These men had been treated for their disease with cisplatin-based chemotherapy regimens, which included cisplatin plus ifosfamide in two cases, when they were 14–20 years of age. Each patient was recruited by in-clinic or mail recruitment protocols. Male survivors were eligible if they met the following criteria: alive, with no evidence of disease; diagnosed with bone or soft tissue sarcoma; off all cancer treatment, including radiation treatment, for at least 2 years; at least 15 years of age at study entry; less than 21 at diagnosis; had received cisplatin as part of cancer treatment; must not have received any other alkylating agent (Cyclophosphamide, Melphalan, Busulfan, BCNU, CCNU, Chlorambucil, Nitrogen Mustard, Procarabazine, or Thiotepa); must have received all or part of their cancer treatment at one of the collaborating sites; free of any pre-condition to cancer treatment that could result in infertility; have had no CNS, abdominal, pelvic, or gonadal radiation therapy or total body irradiation (TBI); proficiency in English as designated in patient’s medical record; provided written informed consent or assent, and authorization to access medical records under HIPAA. Note that relapsed patients and patients with a subsequent malignancy (SMN) that are treated with surgery alone for the relapse or SMN were eligible for this study as long as they meet the above criteria. Controls were recruited from among adult men with no history of cancer who had previously participated as controls in the Fred Hutchinson Cancer Research Center, Seattle WA, ATLAS study [22, 23]. These men were re-contacted regarding participation in the current study.

Each patient and control was asked to provide a semen sample via home seminal fluid collection, which we used to allow for ease of subject participation since the sample can be obtained without the individual traveling to a laboratory. Sperm concentration and morphology measures were performed on semen that had undergone liquefication during shipping, consistent with the WHO protocol for semen analysis [24]. For sperm concentration (per ml), each participant’s semen was diluted and assessed by CASA (Computer Assisted Sperm Analysis). Three separate counts were performed and the results averaged. A small volume of semen was washed for making histology smears for morphology assessments, based on 200 sperm. Although sperm motility was not assessed (because it requires a fresh sample), count and morphology data nonetheless provide a great deal of information regarding spermatogenesis and abnormalities and both are associated with an increased risk of infertility [25]. All protocols were approved by the Seattle Children’s Hospital institutional IRB committee (#12839 and 13158).

### DNA Preparation

Frozen human sperm samples were stored at -20 C and thawed for analysis. Genomic DNA from sperm was prepared as follows: One hundred μl of sperm suspension was used then 820 μl DNA extraction buffer (50 mM Tris pH 8, 10 mM EDTA pH 8, 0.5% SDS) and 80 μl 0.1 M Dithiothreitol (DTT) added and the sample incubated at 65 C for 15 minutes. 80 μl Proteinase K (20 mg/ml) was added and the sample incubated on a rotator at 55 C for 2 hours. After incubation, 300 μl of protein precipitation solution (Promega, A795A, Madison, WI) was added, the sample mixed and incubated on ice for 15 minutes, then spun at 4 C at 13,000 rpm for 20 minutes. The supernatant was transferred to a fresh tube, then precipitated over night with the same volume 100% isopropanol and 2 μl glycoblue at -20 C. The sample was then centrifuged and the pellet washed with 75% ethanol, then air-dried and resuspended in 100 μl H2O. DNA concentration was measured using the Nanodrop (Thermo Fisher, Waltham, MA). The freeze-thaw and subsequent sonication will destroy any contaminating somatic cells within the sperm collection.

### Methylated DNA Immunoprecipitation (MeDIP)

Methylated DNA Immunoprecipitation (MeDIP) with genomic DNA was performed as previously described and optimized with H19 gene [8] as follows: Human sperm DNA pools were generated by combining 2 μg of genomic DNA from each individual. Pooling was performed to maintain inter-individual variation and reduce cost as previously described [14]. With 9 individuals per group, a total of 6 pools were created (3 pools each of control and chemotherapy exposed subjects) and labeled human sperm pools (HS#) #1 through #6. The resulting 6 μg of genomic DNA per pool was diluted to 150 μl with 1x Tris-EDTA (TE, 10 mM Tris, 1 mM EDTA) and sonicated with a probe sonicator using 5 x 20 pulses at 20% amplitude. Fragment size (200–800 bp) was verified on a 1.5% agarose gel. Sonicated DNA was diluted to 400 μl with 1xTE and heated to 95 C for 10 minutes, then incubated in ice water for 10 minutes. Then 100 μl of 5 x immunoprecipitation (IP) buffer (50 mM Sodium Phosphate pH 7, 700 mM NaCl, 0.25% Triton X-100) and 5 μg of 5-mC monoclonal antibody (Diagenode, Denville, NJ, C15200006-500) were added and the sample incubated on a rotator at 4 C over night. The next day Protein A/G Agarose Beads from Santa Cruz were prewashed with 1xPBS/0.1% BSA and resuspended in 1 x IP buffer. Eighty μl of the bead slurry were added to each sample and incubated at 4 C for 2 hours on a rotator. The bead-DNA-antibody complex was washed 3 times with 1 x IP buffer by centrifuging at 6,000 rpm for 2 minutes and resuspending in 1 x IP buffer. After the last wash the bead-complex was resuspended in 250 μl of digestion buffer (50 mM Tris pH 8, 10 mM EDTA pH 8, 0.5% SDS) with 3.5 μl Proteinase K (20mg/ml) per sample and incubated on a rotator at 55 C for 2 hours. After incubation DNA was extracted with the same volume of Phenol-Chloroform-Isoamyalcohol and then with the same volume chloroform. To the supernatant from chloroform extraction 2 μl glycoblue, 20 μl 5M Sodium Chloride and 500 μl 100% cold ethanol were added. DNA was precipitated at -20 C over night, then spun for 20 minutes at 13,000 rpm at 4 C, washed with 75% ethanol and air-dried. Dry pellet was resuspended in 20 μl H2O and concentration measured in Qubit using the Qubit ssDNA Assay Kit (Life Technologies, Carlsbad, CA).

### MeDIP-Seq Analysis

The MeDIP pools were used to create libraries for next generation sequencing (NGS) at the University of Reno, NV Genomics Core Laboratory using the NEBNext® Ultra™ RNA Library Prep Kit for Illumina® (San Diego, CA) starting at step 1.4 of the manufacturer’s protocol to generate double stranded DNA. After this step the manufacturer’s protocol was followed. Each pool received a separate index primer. NGS was performed at that same laboratory using the Illumina HiSeq 2500 with a PE50 application, with a read size of approximately 50bp and approximately 100 million reads per pool. Two libraries each were run in one lane comparing one control with one chemotherapy exposed pool in each lane.

A partial validation of the MeDIP-Seq analysis used a bisulfite sequencing analysis involving approximately 200 million reads for all samples combined for chemotherapy and control groups. The genomic DNA was pooled and treated with bisulfite [26] then the DNA libraries made and sequenced on an Illumina 2500 platform and the sequence used to determine the CpG C to T conversion rates of a multiple adjacent site DMR located on chromosome 3. The combined sites had greater than 100 reads per CpG site. This information was used to identify DNA methylation of specific CpG sites of the chromosome 3 DMR. The chemotherapy and control samples were analyzed and compared to validate the differential DNA methylation of the DMR CpG sites with the MeDIP-Seq identification of the DMR.

### CNV-Seq Analysis

Genomic DNA extracted from sperm was used to create pools containing the same individuals as used for MeDIP-seq. Equal amounts of each individual’s genomic DNA were used for each pool with a final amount of 2 μg per pool. The pools were diluted to 130 μl with 1 x TE buffer and sonicated in a Covaris M220 with the manufacturer’s preset program to create fragments with a peak at 300 bp. Aliquots of the pools were run on a 1.5% agarose gel to confirm fragmentation. The NEBNext DNA Library Kit for Illumina was used to create libraries for each pool, with each pool receiving a separate index primer. The libraries were sent to the WSU Genomics Core in Spokane, WA for NGS on the Illumina HiSeq 2500 using a PE50 application. All 6 libraries were run in one lane and comparisons were performed. Approximately 30 million reads were obtained for each sample for comparison.

### Bioinformatics and Statistics

Basic read quality was verified using summaries produced by the FastQC program [27]. The reads for each sample for both CNV and DMR analyses were mapped to the GRCh38 human genome using Bowtie2 [28] with default parameter options. The mapped read files were then converted to sorted BAM files using SAMtools [29]. To identify DMR, the reference genome was broken into 100 bp windows. The MEDIPS R package [30] was used to calculate differential coverage between control and exposure sample groups. The edgeR p-value [31] was used to determine the relative difference between the two groups for each genomic window. Windows with an edgeR p-value less than 10^−4^ were considered DMRs. The DMR edges were extended until no genomic window with an edgeR p-value less than 0.1 remained within 1000 bp of the DMR. CpG density and other information was then calculated for the DMR based on the reference genome. The DMRs that included at least two windows with an edgeR p-value <10^−4^ were then selected for further analysis and annotated.

The bisulfite reads were first cleaned using Trimmomatic [32]. The reads were then mapped to the GRCh38 human genome using Bismark [33]. Regions of the genome corresponding to DMR identified with MeDIP-seq were extracted for manual review. A single DMR was identified with sufficient read depth. This is likely due to the presence of a repeat element within the DMR. A single representative of the repeat element was extracted and used as a reference sequence. The bisulfite reads overlapping with the DMR were then remapped to this element using Bowtie2 (with parameters–D 10 -R 5 -N 1 -L 10—local–mp 2). All reads from control pools were then compared with all reads from treatment pools to identify CpG sites with differential C/T conversion.

The cn.MOPS R package [34] was used to identify potential copy number variation (CNV). The cn.MOPS analysis detects CNVs by modeling read depth across all samples. The window size used by the cn.MOPS analysis was chosen dynamically for each chromosome based on the read coverage. For chromosomes 1 to 22 the window size ranged from 10 kb to 20 kb. For the mitochondrial, X, and Y chromosomes the window sizes were 1 kb, 31 kb, and 42 kb, respectively. We considered only CNV that occurred exclusively in either all control or all treatment samples.

DMR clusters were identified with R script (www.skinner.wsu.edu under genomic data) using a 2 Mb sliding window with 50 kb intervals. DMR were annotated using the biomaRt R package [35] to access the Ensembl database [36]. The genes that overlapped with DMR were then input into the KEGG pathway search [37, 38] to identify associated pathways. The DMR associated genes were manually then sorted into functional groups by consulting information provided by the DAVID [39], Panther [40], and Uniprot databases incorporated into an internal curated database (www.skinner.wsu.edu under genomic data). All MeDIP-Seq genomic data obtained in the current study have been deposited in the NCBI public GEO database (GEO #: GSE85790).

## Results

### Patient and Sperm Collection

Characteristics of the chemotherapy-exposed patients and controls including age of semen collection, specific chemotherapy and sperm quality are presented in S1 Table. The age of exposure ranged 14 yr. to 20 yr. Upon collection the sperm numbers ranged from 7 to 518 million total with the control population mean of 280 million total per individual and the chemotherapy-exposed mean of 77.8 million total per individual. Therefore, there was a general reduction in sperm number in the chemotherapy-exposed population, as previously described [2, 3], but the patients had approximately 30% normal sperm counts. Chemotherapy exposed men had, on average, a slightly higher percentage of sperm with abnormal morphology than controls. This difference was primarily due to differences in non-head defects, S1 Table.

### Epigenetic Analysis of Sperm

The MeDIP-Seq analysis provided a high read number and alignment proportion for each of the six pools (Table 1A). The differential DNA methylation regions (DMRs) were identified using the MEDIPS R package as outlined in the Methods. The DMRs include single statistically significant 100 bp sites as well as multiple (e.g. adjacent) 100 bp sites. The DMRs for all sites and multiple sites are shown for a variety of statistical p-value thresholds in Table 1B. The p<10^−4^ was selected for further analysis to reduce the potential false positives of larger p-values. In addition, due to the higher potential for false positives in single sites the multiple site p<10^−4^ was used for subsequent analysis and discussion. The more variable single sites are likely important, but the more stringently selected multiple site DMRs are used to convey the general observations. Therefore, the 2831 single sites and 135 multiple sites are discussed. The distribution of the DMR according to number of multiple sites is presented in Table 1C and the list of p<10^−4^ multiple site DMRs is shown in S2 Table. The individual DMR characteristics for chromosomal location, size, CpG density, statistics and gene associations are summarized in S2 Table. The majority are single site DMRs with the bulk of the multiple site DMR having two sites. Interestingly, one DMR (DMR3:198096901) had 73 multiple windows on chromosome 3, none of which were associated with a known gene (S2 Table). Further investigation showed that this DMR contains a non-typical repeat DMR element. Observations demonstrate the adolescent chemotherapy exposure induced sperm epimutations in adult males approximately ten years following chemotherapy.

**Table 1:** DMR number and characteristics. **A)** The number of reads present for each sample pool (HS #) and overall alignment rate calculated by bowtie2. **B)** The number of DMRs found using different edgeR p-value cutoff thresholds. **C)** The number of DMR with associated specific number of significant sites at a p-value threshold of <10^−4^.

A. Read number and alignment								
	HS1	HS2	HS3	HS4	HS5	HS6		
Read Number	127916479	116715381	60181475	67042941	78944306	126030851		
Alignment %	97.90	97.77	97.77	97.92	96.19	97.19		
B. DMR number								
**p-value**	**Total DMR**	**Multiple Site DMR**						
0.001	20526	1551						
**1.00E-04**	**2831**	**135**						
1.00E-05	463	24						
1.00E-06	76	6						
1.00E-07	15	2						
C. Site number associated with DMR								
Number of Significant Sites	1	2	3	4	5	6	8	73
Number of DMR	2696	110	13	5	4	1	1	1

**Fig 1:**
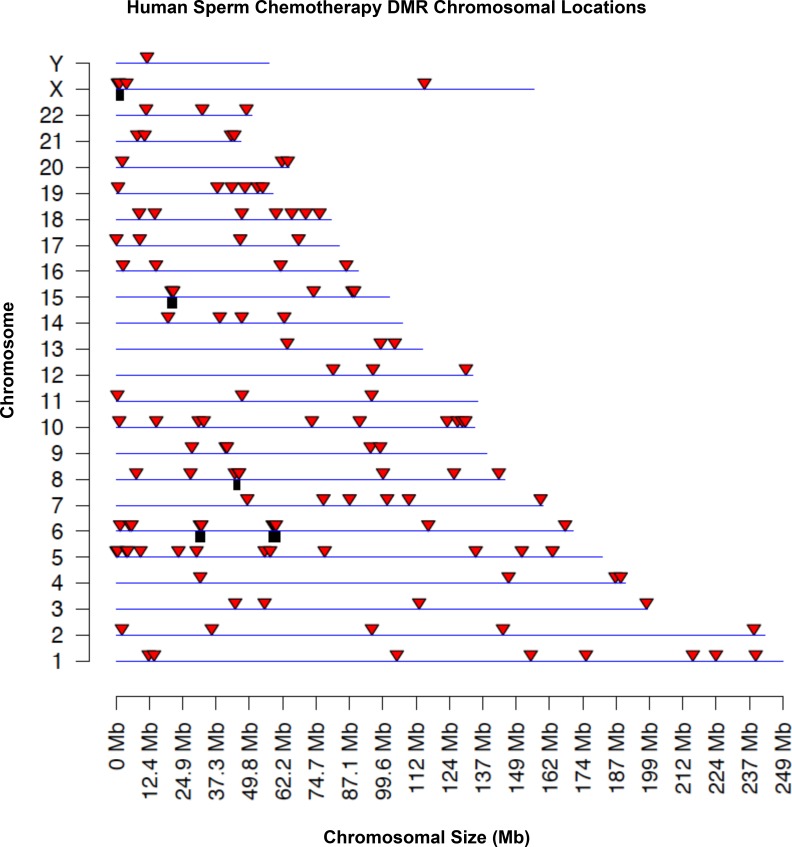
Human sperm chemotherapy-associated DMR chromosome location. The DMR locations on the individual chromosomes are presented as a red arrowhead. Only DMR containing at least two significant sites at a p-value threshold of 1e-04 are shown. The black box under the chromosome line represents statistically significant over-represented clusters of DMR within the chromosomal size of the box.

The chromosomal location of the sperm DMRs/epimutations is presented in Fig 1. The DMRs were present on all chromosomes, including the Y chromosome, with a number of statistically over-represented clusters of DMRs indicated with the black box below the line. This group of DMR is referred to as an epimutation signature derived from the different human sperm pools. As a comparison the chromosomal plot of the single site DMRs is presented in S1 Fig. The single site DMR density was greater, but interestingly several regions in chromosome 1, 9, 13, 14, 15 were void of DMRs. These regions may have structural elements that prevent DMR formation. In addition, a larger number of DMR clusters were observed in all chromosomes (S1 Fig). Although the current study demonstrates a signature of statistically significant epimutations are present in the adolescent chemotherapy-exposure population, future analysis with expanded patient populations is required to determine differential effects between different chemotherapies and periods of developmental exposure.

**Fig 2:**
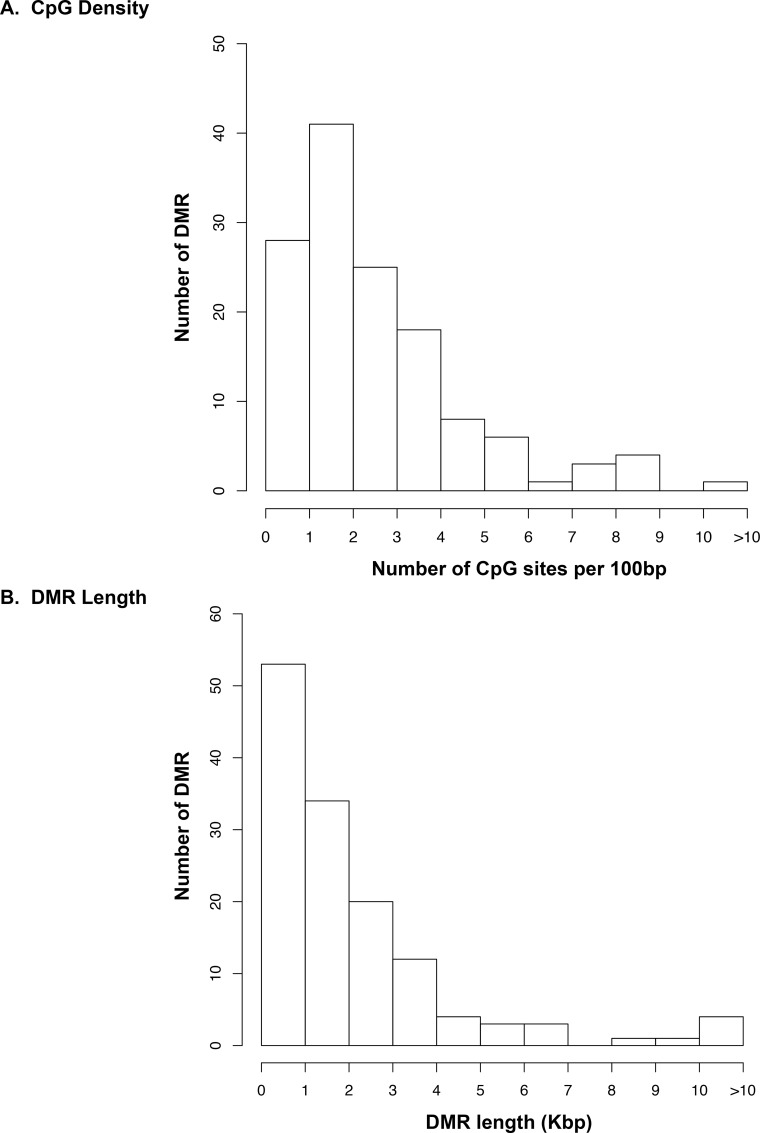
Human sperm chemotherapy-associated DMR genomic feature and size. **A)** The CpG density of the DMR is presented as number of CpG/100bp with the corresponding number of DMR. **B)** The DMR length in kilobase pairs (kb) is presented with the corresponding DMR number. Only DMR containing multiple significant windows at a p-value threshold of 1e-04 are shown.

A genomic feature identified in all previously detected environmentally induced epimutations was a region of low density CpG content termed a CpG desert [41]. Analysis of the CpG content of the chemotherapy-associated human sperm epimutations identified between 1–3 CpG per 100 bp density with only one DMR having a greater than 10 CpG/100 bp (Fig 2A and S2 Table). Therefore, the DMR were in CpG deserts and none identified in CpG islands. The epimutations were predominantly 1 kb in size with only a few greater than 6 kb in size (Fig 2B and S2 Table). Therefore, the genomic features of the human sperm epimutations identified following chemotherapy exposure were similar to those previously identified in other species using a variety of different exposures [41]. This conservation suggests a potential common mechanism with broad impacts on medicine.

A limitation of the current study is the small sample size (total n = 18) used, so additional molecular analysis was performed. The potential that molecular (i.e. epigenetic) variation within each of the groups being compared may contribute to the DMR identified was investigated. Analysis of the internal population variation in the unexposed and exposed populations separately identified 114 and 50 overlapping single site DMR respectively (S2 Fig). The three individual pools of each population were compared between each other to identify the internal population variation in DMR. The majority of internal population variation is anticipated to be hypervariable DMR, termed metastable epialleles [42], and none of these internal population DMR overlapped with each other or with the exposed versus unexposed DMR dataset. Therefore, internal population variation does not account for the chemotherapy associated DMR identified in sperm.

### MeDIP-Seq Validation

The MeDIP-Seq protocol identifies the differential DNA region (DMR) but does not identify the CpG level changes in DNA methylation. A preliminary analysis using bisulfite sequencing was used to examine a selected DMR for CpG level changes and help validate the MeDIP-Seq analysis. Since it is not possible to predict specific CpG methylation changes, a bisulfite-Seq analysis was performed. Due to sample availability and prohibitive cost, a minimal read depth was used. A selected DMR in a repetitive element was used that did provide the required read depth needed. The genomic DNA was bisulfite treated and analyzed with approximately 200 million reads sequenced for all samples combined. Sequencing reads mapping to this DMR were extracted and remapped to a single element of the repeat using Bowtie2. A single DMR containing multiple adjacent sites within a repetitive element on chromosome 3 was selected for analysis. The CpG sites with greater than 100 reads on chromosome 3 were selected and used to identify the bisulfite C to T conversion for a number of DMR CpG sites, as shown in S3A Fig. The C to T conversion rate and percent methylation for the CpG sites are presented and show a difference between the control and chemotherapy samples, S3B Fig. These changes between the chemotherapy and control samples help validate the MeDIP-Seq detection of this DMR.

### Genetic Analysis of Sperm

Analysis of a genetic mutation (copy number variation, CNV) was performed to determine the genetic CNV variation in the exposed versus unexposed comparison. Although variable CNV were detected within the different pools of the populations, S4 Fig, comparison of the exposed versus unexposed populations identified minimal alterations present in all pool comparisons. None of the CNV were associated or overlapped with the DMR identified. Only 3 CNV were found to overlap between the exposed versus unexposed populations. Therefore, genetic CNV variation does not appear to be a cause for the epigenetic differences observed. The genetic CNV variation observed is to be expected within the human population [43] and the increased variation on specific chromosomes, S4 Fig, with higher repeat element percentages is expected.

### Differential DNA Methylation Region (DMR) Gene Associations

**Fig 3:**
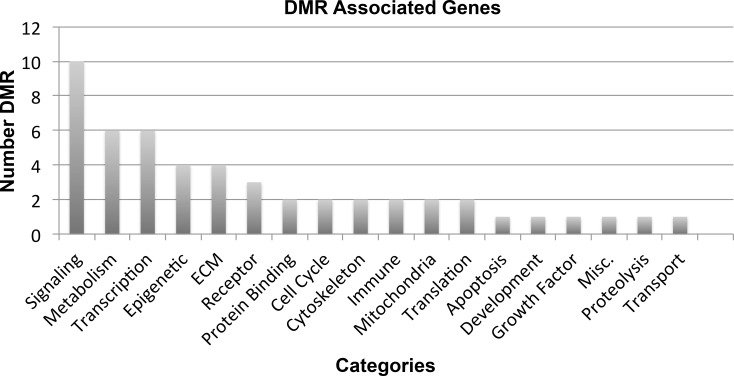
The human sperm chemotherapy-associated DMR associated gene classifications (i.e. functional categories). The number of DMR associated genes for specific classification categories are presented.

The gene associations with the DMRs are listed in Table 2 and the complete list with information in S2 Table and S3 Table. Approximately 50% of the DMRs had associations with genes indicating half the epimutations are intergenic and distal from genes. Previously some DMRs have been suggested to potentially act as epigenetic control regions and distally regulate expression through ncRNA mechanisms for 2–5 Mbase regions [44]. The DMR associated with genes were primarily in predicted promoter regions (10 kb). The genes associated with chemotherapy-associated DMRs are present in numerous gene classifications with no major category being over-represented (Table 2 and S3 Table). The number of DMR associated with specific gene classification categories are presented in Fig 3. The DMR associated genes were analyzed for correlated known gene pathways. No specific pathway or cellular process was found to have more than four associated genes. These results suggest that the chemotherapy induced sperm DMR have the potential to alter genome activity.

**Table 2:** DMR associated gene list and categories. The specific DMR, associated gene symbol and classification category are presented. Some DMR are associated with multiple genes which are listed. DMR associated genes with unknown classification only are not listed.

DMR Name	Gene Association	Category	DMR Name	Gene Association	Category
DMR2:238198201	ILKAP	Signaling	DMR1:175491101	TNR	ECM
DMR5:164501	PLEKHG4B	Signaling	DMR5:151531101	FAT2	ECM
DMR5:55530601	PPAP2A	Signaling	DMR9:28333101	LINGO2	ECM
	RNF138P1	Unknown	DMR19:43206901	PSG4	ECM
DMR5:9122501	SEMA5A	Signaling	DMR1:12173801	TNFRSF1B	Receptor
DMR7:158556901	PTPRN2	Signaling	DMR9:98644001	GABBR2	Receptor
DMR8:99694501	VPS13B	Signaling	DMR10:123691901	GPR26	Receptor
	AC018442.1	Unknown	DMR6:31814701	HSPA1L	Protein Binding
DMR13:98815001	DOCK9	Signaling		HSPA1A	Protein Binding
DMR16:2603101	PDPK1	Signaling	DMR6:31828001	HSPA1B	Protein Binding
	AC141586.5	Unknown	DMR5:134314001	CDKL3	Cell Cycle
DMR17:8836901	PIK3R6	Signaling		CTD-2410N18.4	Unknown
DMR18:8634901	RAB12	Signaling	DMR19:756801	MISP	Cell Cycle
DMR10:73117601	NUDT13	Metabolism	DMR18:46969701	KATNAL2	Cytoskeleton
DMR11:484301	PTDSS2	Metabolism		TCEB3CL	Transcription
DMR12:81062701	ACSS3	Metabolism	DMRX:115191101	LRCH2	Cytoskeleton
DMR12:95948901	AMDHD1	Metabolism		RBMXL3	Translation
DMR14:46935601	MDGA2	Metabolism	DMR6:31326001	HLA-C	Immune
DMR20:2311001	TGM3	Metabolism	DMR19:54772001	KIR2DL1	Immune
DMR2:2189101	MYT1L	Transcription		KIR3DL1	Immune
DMR2:144492201	ZEB2	Transcription		CTB-61M7.1	Unknown
DMR10:30846501	ZNF438	Transcription	DMR7:87205701	TMEM243	Mitochondria
DMR10:32731701	CCDC7	Transcription	DMR7:101239401	FIS1	Mitochondria
DMR19:52916701	ZNF888	Transcription	DMR10:1197701	ADARB2	Translation
DMR20:61964901	TAF4	Transcription	DMR22:11248401	5_8S_rRNA	Translation
DMR4:186476501	F11-AS1	Epigenetic		AC137488.1	Unknown
	RP11-215A19.2	Unknown	DMR19:48181401	CARD8	Apoptosis
DMR4:188443001	LINC01060	Epigenetic		ZNF114	Transcription
DMR8:27797501	ESCO2	Epigenetic		C19orf68	Unknown
DMR16:14910901	MIR3180-1	Epigenetic	DMR3:55470001	WNT5A	Development
	NPIPA3	Unknown	DMR22:48701801	FAM19A5	Growth Factor
	RP11-958N24.1	Unknown	DMR12:130657401	RIMBP2	Misc.
	NPIPA1	Unknown		RP11-662M24.2	Unknown
				RP11-662M24.1	Unknown
			DMR9:95044801	NPEPO	Proteolysis
			DMR14:62802301	KCNH5	Transport

## Discussion

Our observations suggest that altered sperm DNA methylation can develop from early life cancer chemotherapy exposure and may correlate to alterations in sperm morphology, number and ultimately male fertility. Although other epigenetic changes could also be involved, DNA methylation has been shown to have more developmental and genome wide influences than many of the other epigenetic factors [45]. To our knowledge, this study is the first examination of the actions of current chemotherapy regimens on the human sperm epigenome and spermatogenesis. Previous studies have suggested no evidence in humans of adverse effects of chemotherapy treatment in offspring (less than five years of age) of male cancer survivors [46–48]. However, at the time of these studies, many male survivors had not yet attempted to sire a pregnancy and the number of pregnancies from partners of male survivors were small. No studies have examined later life adult and potentially generational impacts.

Previous studies in non-human model systems have demonstrated that a variety of exposures can promote epigenetic alterations in the germline [6, 7, 14, 49–51]. Environmental toxicants including the fungicide vinclozolin [7, 8], pesticides DDT and methoxychlor [7], plastic derived compounds BPA and phthalates [15], and hydrocarbons [16] can promote altered epigenetic (DNA methylation) programming in sperm [14]. Although a previous mouse study demonstrated chemotherapy promoted generational disease phenotypes with a genetics interpretation [19], the ability of environmental exposures to promote sperm epimutations suggested to us that chemotherapy may also promote altered germ cell epigenetic programming in humans. The current study was designed to investigate the effects of adolescent chemotherapy exposure on later life adult sperm epimutations. Our results demonstrate the presence of DMRs or epimutations in the sperm of men that had adolescent chemotherapy exposure. The patients were treated for osteosarcoma, not testicular cancer. Approximately a decade had passed since the cancer patients’ chemotherapy. More advanced spermatogenic cells would have been lost after 100 days following chemotherapy due to the developmental period of the spermatogenic cells in the testis. The observation of epigenetic alterations in the sperm long after chemotherapy strongly suggests that the spermatogonial stem cells in the testis had a permanent epigenetic alteration such that the adult male will produce sperm with epimutations throughout life. Our analysis and selection under high stringency (i.e. multiple site DMR with p<10^−4^) identified a group (i.e. signature) of sperm epimutations associated with chemotherapy exposed individuals. The lower stringency single site DMRs identified are more variable between individuals, but also reflect chemotherapy exposure associated DMR. Clearly future studies with expanded populations are needed to further understand this phenomenon, but the presence of a significant epimutation chemotherapy signature demonstrates the ability of early life chemotherapy to promote germline epimutations. Although the age range of the control and exposure populations had some overlap, the mean age of 25 yr for exposed and 36 yr for unexposed was different. Previous studies demonstrate no major epigenetic differences between these young 25 and 35 ages, but differences are observed with more advanced ages [52]. Studies to demonstrate the epigenetic changes with greater age differences identified associated genes and none of those previously identified genes overlapped with the DMR (multiple window p<10^−4^) associated genes in the current study [52, 53]. The current study was designed to examine adolescent (i.e. pubertal) male exposure, however, since the same populations of spermatogenic stem cells are present throughout adult life, potential chemotherapy induced sperm epimutations may occur any time a male is exposed to chemotherapy. Therefore, the cryopreservation of gametes prior to chemotherapy may be important for patients and their oncologists to consider in the future [4].

The sperm epimutations identified were present on all chromosomes with a number being clustered in statistically significant over-represented groups of DMR. The clustering of DMR is speculated to represent critical regulatory regions within epigenetic control regions [44]. Interestingly, the genomic features of these human sperm chemotherapy associated epigenetics were similar to previously identified sperm epimutations. In particular, one of the major genomic features is a low density CpG content within the DMR referred to as a CpG desert [41]. The CpG density was less than ten percent and the mean was around two CpG/100bp. Due to the evolutionary conservation of these CpG clusters in a CpG desert they are speculated to be regulatory sites [41]. No DMR were identified that associated with CpG islands or shores.

The selection of DMR was focused on multiple site DMR with a high statistical significance. Although a higher rate of false positives is anticipated in the much more common single site DMRs, these single sites are anticipated to be an important component of the chemotherapy induced sperm epimutations. Expanded studies are needed to further investigate the epimutation profiles in the sperm and the physiological impacts. The degree of internal population DMR variation and genetic CNV variation indicated negligible impact on the DMR detected. A large proportion of the epimutations identified were found to have gene associations. No predominant pathways or cellular processes appear over-represented by the epimutation associated genes. Previous studies have demonstrated the ability of DMR/epimutations to cause altered somatic cell gene expression [44]. Therefore transmission of the sperm epimutations to the subsequent generation may alter somatic cell gene activity in offspring.

The germline (e.g. sperm) transmission of epigenetic information can promote the epigenetic transgenerational inheritance of disease and phenotypic variation [12, 13, 44]. A variety of environmental factors from nutrition to toxicants have been shown in a variety of species from plants to humans to promote the epigenetic transgenerational inheritance phenomenon [6]. Since epigenetic inheritance requires the germline (egg or sperm) transmission of epigenetic information between generations [6, 9–11, 49, 50], the alterations of epigenetic processes in the germline need to be established. Developmentally the DNA methylation is erased after fertilization to create the embryonic stem cell totipotency, which then is remethylated in a cell specific manner during embryonic development [54]. Therefore, the majority of the DNA methylation is reset upon fertilization and during primordial germ cell development of the germline [54, 55]. However, a set of genes termed imprinted genes are protected from DNA methylation erasure at fertilization allowing them to be transmitted transgenerationally [6]. No imprinted genes were identified in the DMR (multiple window p<10^−4^) associated genes identified. In the event an environmental exposure modified the epigenetic programming of the germline (e.g. sperm) and these sites become imprinted-like they can promote the epigenetic transgenerational inheritance of disease [6, 56]. Previous studies have documented the ability of caloric restriction to induce the epigenetic inheritance of disease in humans [11, 57]. The current study identifies the ability of chemotherapy to reprogram the epigenome of human sperm. These epimutations can be transmitted to the developing embryo of the next generation. In the event these are imprinted-like epimutations then they would not be erased and would potentially promote the epigenetic transgenerational inheritance to subsequent generations. Future studies are required to investigate the ability of chemotherapy to induce epigenetic inheritance to subsequent generations in humans. The current study suggests the potential for such a phenomenon.

## Conclusions

In summary, the current study demonstrates for the first time the ability of chemotherapy to promote epigenetic reprogramming in the spermatogonial stem cell population that will lead to human sperm epimutations later in life. These DMRs have some gene associations that could influence genome activity. A set of epimutations (i.e. signature) was detected and may provide an epigenetic biomarker for chemotherapy exposures. The potential biological impact of chemotherapy induced epimutations may be to transmit altered epigenetic information to the next generation and if imprinted-like to subsequent generations progeny. The generational impact of the chemotherapy induced germline (i.e. sperm) epimutations needs to be assessed in future studies, but the current study suggests the potential of such a phenomenon. Therefore, the impact of chemotherapy on subsequent generations needs to be assessed, but has not been previously considered outside the realm of induced genetic mutations [58]. Since other environmental exposures do have the ability to promote the epigenetic transgenerational inheritance of disease, the potential generational impacts of chemotherapy need to be investigated.

## Supporting Information

S1 Fig The human sperm chemotherapy-associated all site DMR locations on the individual chromosomes is presented. All site (single and multiple site) DMR are represented with a red arrowhead and the DMR clusters with a black box. All site DMRs at a p-value threshold of 1e-04 are shown.
(PDF)

S2 Fig MeDIP pairwise analysis internal population epigenetic variation. The analysis presenting the DMR numbers is presented and Venn diagrams showing the pairwise overlap of the DMR.
(PDF)

S3 Fig Validation of CpG methylation of DMR3:198096901 using bisulfite sequencing analysis. The chromosome 3 DMR specific CpG are shown in the sequence **(A)**. The conversion of C to T for Control versus Chemotherapy is shown in **(B)**. The percentage CpG methylation sites presented in **(C)**.
(PDF)

S4 Fig CNV analysis summary for the human sperm. The non-exposed (HS1, HS2, HS3) control and chemotherapy exposed (HS4, HS5, HS6) population pools are listed.
(PDF)

S1 Table Patient Information **A)** Information for the chemotherapy-treated and control individuals with treatment age, sperm collection age, chemotherapy used and total sperm number presented. **B)** Average and range for the chemotherapy-treated patients and controls presented for age at collection, age at treatment, cisplatin dose (milligrams/meter squared), ifosfamide dose and seminal fluid volume. Note that one patient that got 120 mg/m2 dose of cisplatin also got 800 mg/m2 of carboplatin which is in the same class of drugs as cisplatin. The B006 chemotherapy case sperm count was not determined (ND). NA indicates not applicable.
(PDF)

S2 Table Human sperm chemotherapy-associated DMR list for multiple site DMR at p<10^−4^. The DMR name, chromosomal location, start site, length in base pair (bp), CpG density (CpG/100bp), and gene associated is listed. The absence (not applicable, NA) of one or more gene listed under “Gene Association” indicates an intergenic DMR location.
(PDF)

S3 Table Human sperm chemotherapy-associated DMR associated genes. The DMR name, gene symbol, chromosome location start and end position, Ensembl number, gene description and classification category are presented.
(PDF)

## Abbreviations

AYA: adolescent and young adult
CNV: copy number variation
DDT: dithiothreitol
DMR: differential DNA methylation region
IP: immunoprecipitation
MeDIP: methylated DNA immunoprecipitation
NGS: next generation sequencing
SMN: subsequent malignancy
TBI: total body irradiation
