# Supplementary figures and images for: Differential DNA Methylation Regions in Adult Human Sperm following Adolescent Chemotherapy: Potential for Epigenetic Inheritance
Source: PLoS One. 2017 Feb 1;12(2):e0170085. doi: 10.1371/journal.pone.0170085 (PMC5287489; doi:10.1371/journal.pone.0170085)

Human Sperm Chemotherapy Associated All DMR Chromosomal Locations

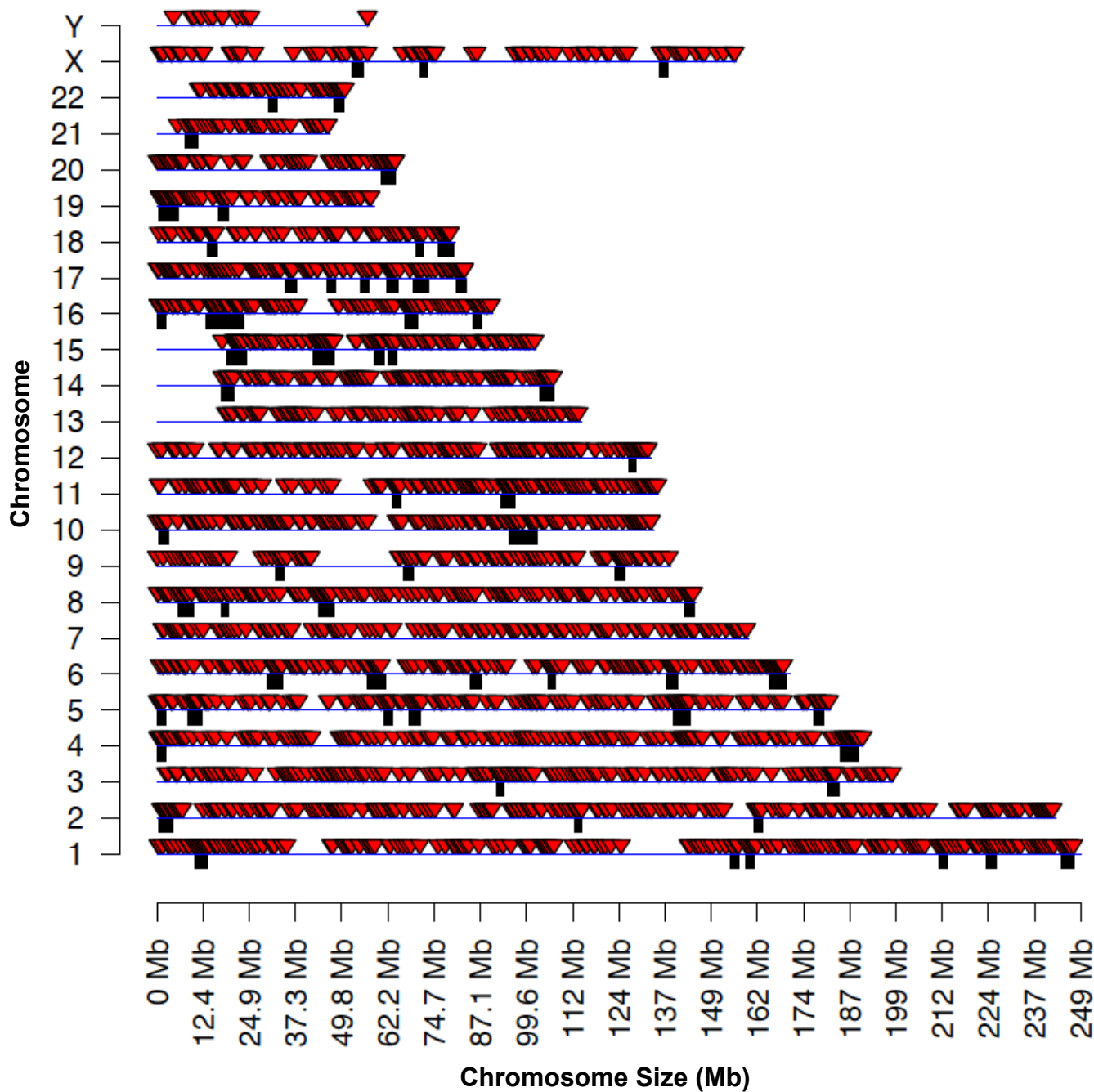

Supplement: S1 Fig — All site (single and multiple site) DMR are represented with a red arrowhead and the DMR clusters with a black box. All site DMRs at a p-value threshold of 1e-04 are shown. (PDF) [file pone.0170085.s001.pdf]
