# Supplementary material for: Differential DNA Methylation Regions in Adult Human Sperm following Adolescent Chemotherapy: Potential for Epigenetic Inheritance
Source: PLoS One. 2017 Feb 1;12(2):e0170085. doi: 10.1371/journal.pone.0170085 (PMC5287489; doi:10.1371/journal.pone.0170085)
Supplement: S2 Fig — The analysis presenting the DMR numbers is presented and Venn diagrams showing the pairwise overlap of the DMR. (PDF) [file pone.0170085.s002.pdf]

Supplemental Figure S2

MeDIP pairwise analysis internal population epigenetic variation

Analysis

The Standard MeDIP analysis pipeline was run to identify regions of differential coverage between pairs of samples. Each sample was compared with every other sample. DMR were identified using an edgeR p-value threshold of 10^{-4}.

The number of DMR found for each analysis. All DMR have at least two significant windows at a p-value threshold of 1e-04. The different pools of 3 individuals each for unexposed, control (C1, C2, C3), and chemotherapy exposed treatment (T1, T2, T3), patients.

Full Analysis	C1vsC2	C1vsC3	C2vsC3	T1vsT2	T1vsT3	T2vsT3
135	28013	11782	433	1468	3438	1175

Overlapping DMR

The number of overlapping DMR between each pairwise analysis and the full analysis

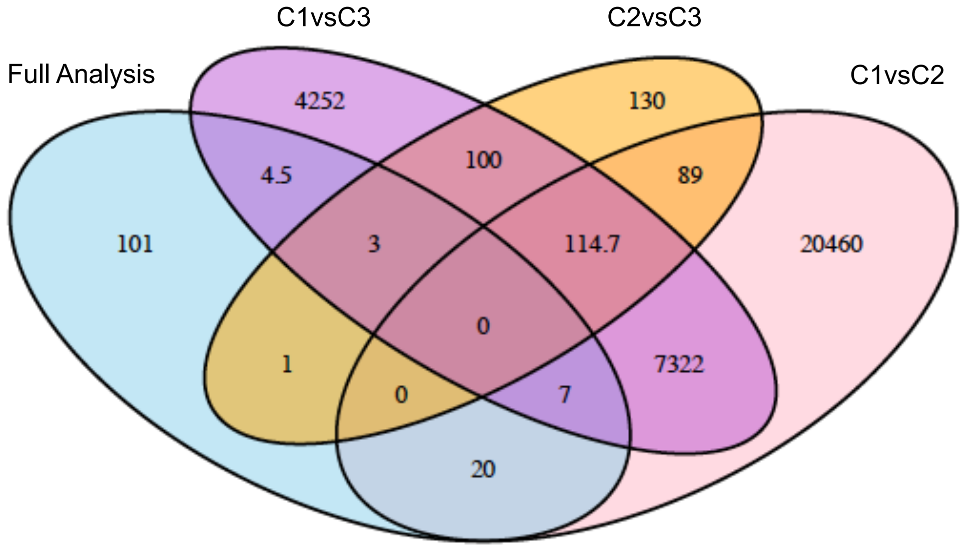

The overlapping DMR between the control vs. control pairwise analyses and the full analysis.

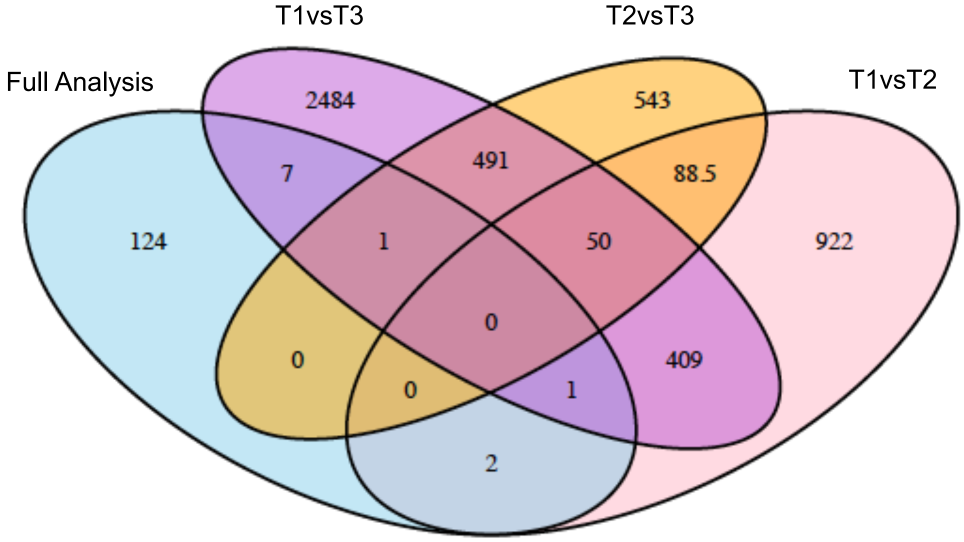

The overlapping DMR between the treatment vs. treatment pairwise analyses and the full analysis.
