# Supplementary material for: Differential DNA Methylation Regions in Adult Human Sperm following Adolescent Chemotherapy: Potential for Epigenetic Inheritance
Source: PLoS One. 2017 Feb 1;12(2):e0170085. doi: 10.1371/journal.pone.0170085 (PMC5287489; doi:10.1371/journal.pone.0170085)
Supplement: S3 Fig — The chromosome 3 DMR specific CpG are shown in the sequence (A). The conversion of C to T for Control versus Chemotherapy is shown in (B). The percentage CpG methylation sites presented in (C). (PDF) [file pone.0170085.s003.pdf]

Supplemental Figure S3

(A) Chromosome 3 DMR CpG Site Sequence

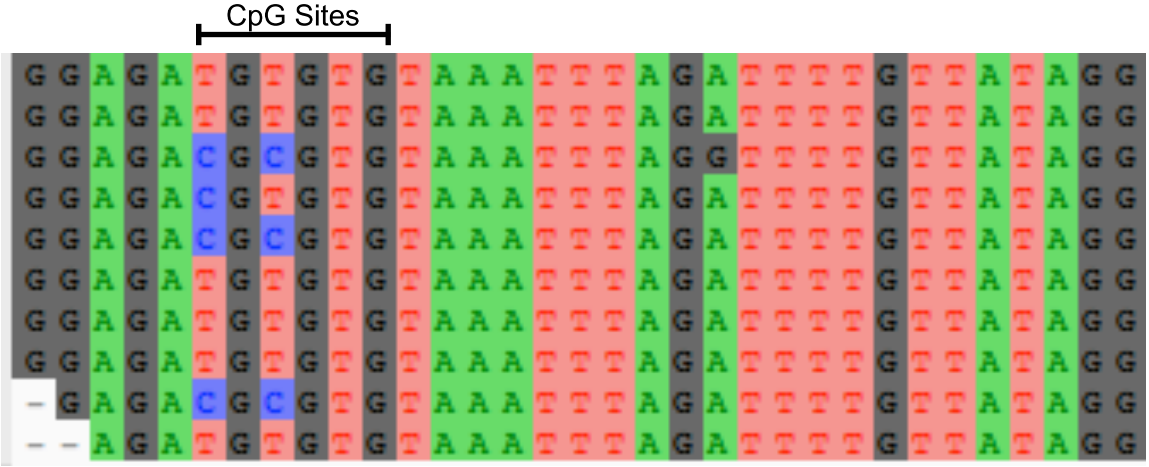

(B) Bisulfite Sequencing Raw Read Counts C to T Conversion

Chemotherapy

Reference	C	G	C	G	T	G
A	0	0	0	21	0	0
C	5	0	2	0	0	0
G	0	104	0	84	0	108
T	100	1	103	2	108	0

Control

Reference	C	G	C	G	T	G
A	1	2	1	124	1	1
C	331	0	272	0	0	0
G	1	601	2	472	0	616
T	271	5	334	18	616	0

(C) CpG Methylation Percentage

Chemotherapy:

CpG 1 95% Methylation

CpG 2 98% Methylation

Control:

CpG 1 55% Methylation

CpG 2 45% Methylation
