# Supplementary material for: Differential DNA Methylation Regions in Adult Human Sperm following Adolescent Chemotherapy: Potential for Epigenetic Inheritance
Source: PLoS One. 2017 Feb 1;12(2):e0170085. doi: 10.1371/journal.pone.0170085 (PMC5287489; doi:10.1371/journal.pone.0170085)
Supplement: S4 Fig — The non-exposed (HS1, HS2, HS3) control and chemotherapy exposed (HS4, HS5, HS6) population pools are listed. (PDF) [file pone.0170085.s004.pdf]

Supplemental Figure S4

CNV analysis summary for the human sperm

Read Mapping Summary:

	HS1	HS2	HS3	HS4	HS5	HS6
Read Number	32352587	27963740	32524539	22628164	30272531	36013163
Overall Alignment Rate	97.41%	78.89%	96.76%	93.03%	86.87%	94.47%

The number of reads present for each sample and the overall alignment rate calculated by bowtie2. The different human sperm pools (HS#) are presented.

Overall CNV Numbers:

	**1**	**2**	**3**	**4**	**5**	**6**	**7**	**8**	**9**	**10**	**11**	**12**
**HS1**	7	4	7	8	5	3	8	5	12	1	14	4
**HS2**	84	68	61	30	54	34	57	38	44	24	57	61
**HS3**	110	77	65	50	53	39	43	46	21	48	35	22
**HS4**	3	0	1	1	6	0	0	2	1	1	1	0
**HS5**	5	1	2	1	1	0	1	1	4	3	3	2
**HS6**	10	5	6	8	7	3	13	6	9	4	15	6

	**13**	**14**	**15**	**16**	**17**	**18**	**19**	**20**	**21**	**22**	**X**	**Y**
**HS1**	3	7	0	2	0	0	0	4	5	0	2	0
**HS2**	44	44	36	35	0	14	2	20	23	25	10	8
**HS3**	26	34	17	41	43	23	33	24	18	21	14	0
**HS4**	2	0	0	5	4	0	12	3	3	1	0	0
**HS5**	0	2	2	0	0	0	0	4	1	2	0	0
**HS6**	3	7	0	12	4	0	0	5	5	2	1	0

The number of CNV found, separated by sample pool (HS#) and chromosome.

Overlapping CNV between control and chemotherapy exposed populations of individuals: 3 CNV
