# Supplementary material for: Differential DNA Methylation Regions in Adult Human Sperm following Adolescent Chemotherapy: Potential for Epigenetic Inheritance
Source: PLoS One. 2017 Feb 1;12(2):e0170085. doi: 10.1371/journal.pone.0170085 (PMC5287489; doi:10.1371/journal.pone.0170085)
Supplement: S1 Table — Patient Information A) Information for the chemotherapy-treated and control individuals with treatment age, sperm collection age, chemotherapy used and total sperm number presented. B) Average and range for the chemotherapy-treated patients and controls presented for age at collection, age at treatment, cisplatin dose (milligrams/meter squared), ifosfamide dose and seminal fluid volume. Note that one patient that got 120 mg/m2 dose of cisplatin also got 800 mg/m2 of carboplatin which is in the same class of drugs as cisplatin. The B006 chemotherapy case sperm count was not determined (ND). NA indicates not applicable. (PDF) [file pone.0170085.s005.pdf]

Supplemental Table S1. Patient Information and Fertility (Sperm)

(A)

ID #	Chemotherapy	Collection Age (yr)	Sperm # (Total Millions)	Pool #
CIS-051	N/A	40	240.5	1
CIS-056	N/A	38	303.6	1
CIS-061	N/A	32	44.2	1
CIS-063	N/A	33	675	2
CIS-067	N/A	39	262	2
CIS-073	N/A	37	280.4	2
CIS-068	N/A	33	21	3
CIS-072	N/A	40	181.2	3
CIS-074	N/A	37	518	3
B006-249	Cisplatin / Ifosfamide	27	ND*	4
CIS-002	Cisplatin	24	100.2	4
CIS-025	Cisplatin	26	170.5	4
CIS-028	Cisplatin	26	178	5
CIS-034	Cisplatin	24	7.34	5
CIS-043	Cisplatin / Ifosfamide	30	42	5
CIS-033	Cisplatin	25	21	6
CIS-044	Cisplatin	19	49	6
CIS-046	Cisplatin	24	55	6

*ND indicates not determined

(B)

	Chemotherapy Cases	Controls
Average (Mean) Age at sample	24.78	36.01
Age Range at Sample	19.12 to 29.86	27.5 to 44.4
Average (Mean) Age at Treatment	16.3	NA
Age Range at Treatment	14.47 to 19.63	NA
Average Cisplatin Dose mg/m^2	389.33 mg/m^2	NA
Cisplatin Dose Range mg/m^2	120* mg/m^2 to 480 mg/m^2	NA
Average Ifosfamide Dose mg/m^2	30.5 mg/m^2	NA
Range Ifosfamide Dose mg/m^2	30.5 mg/m^2 to 30.5 mg/m^2	NA
Seminal Fluid Volume - Average	2.23 ml	2.41 ml
Seminal Fluid Volume - Range	0.6 to 5.0	0.3 to 7.5
Individuals with Normal SF parameters	6 out of 9	6 out of 9
Sperm Morphology	1 normal	1 normal
Sperm Abnormal Morphology – Average (%)	90.6	91.7
% head defect – Average*	90.6	91.3
% tail defect – Average*	14.6	10.1
% midplace / neck defect – Average*	6.8	4.7
% cytoplasm defect – Average*	3.0	1.9

(*) Indicates those subjects with abnormal morphology
