# Supplementary material for: Differential DNA Methylation Regions in Adult Human Sperm following Adolescent Chemotherapy: Potential for Epigenetic Inheritance
Source: PLoS One. 2017 Feb 1;12(2):e0170085. doi: 10.1371/journal.pone.0170085 (PMC5287489; doi:10.1371/journal.pone.0170085)
Supplement: S2 Table — The DMR name, chromosomal location, start site, length in base pair (bp), CpG density (CpG/100bp), and gene associated is listed. The absence (not applicable, NA) of one or more gene listed under “Gene Association” indicates an intergenic DMR location. (PDF) [file pone.0170085.s006.pdf]

Supplemental Table S2 Multiple Site DMR List

DMR Name	Chr	Start	Length (bp)	Windows	min P Value	CpG #	CpG Density (#/100bp)	Gene Association
DMR1:12173801	1	12173801	1400	2	4.08E-05	27	1.9	TNFRSF1B
DMR1:14192301	1	14192301	900	2	5.38E-05	6	0.6	
DMR1:104846301	1	104846301	2500	2	5.56E-05	26	1	
DMR1:154878801	1	154878801	700	2	1.08E-05	22	3.1	
DMR1:175491101	1	175491101	3200	2	1.49E-05	47	1.4	TNR
DMR1:215393901	1	215393901	500	2	6.42E-05	26	5.2	RP11-199H2.2
DMR1:224010101	1	224010101	4700	2	9.49E-06	108	2.2	RP11-504P24.3
DMR1:238863501	1	238863501	2100	2	1.33E-07	8	0.3	
DMR2:2189101	2	2189101	1100	4	6.30E-07	35	3.1	MYT1L
DMR2:35752601	2	35752601	4600	2	8.10E-06	24	0.5	
DMR2:95550301	2	95550301	1300	2	3.14E-06	29	2.2	
DMR2:144492201	2	144492201	1400	2	1.52E-06	11	0.7	ZEB2
DMR2:238198201	2	238198201	1100	2	3.28E-06	8	0.7	ILKAP
DMR3:44495901	3	44495901	1400	2	4.11E-05	21	1.5	
DMR3:55470001	3	55470001	1000	2	7.95E-07	35	3.5	WNT5A
DMR3:113198301	3	113198301	200	2	4.33E-05	2	1	
DMR3:198096901	3	198096901	10900	73	9.27E-30	397	3.6	
DMR4:31437301	4	31437301	2600	2	9.28E-06	51	1.9	
DMR4:146592201	4	146592201	400	2	1.71E-05	2	0.5	
DMR4:186476501	4	186476501	1900	2	8.09E-06	17	0.8	F11-AS1;RP11-215A19.2
DMR4:188443001	4	188443001	6600	2	1.70E-06	99	1.5	LINC01060
DMR5:164501	5	164501	2200	3	8.79E-06	142	6.4	PLEKHG4B
DMR5:561701	5	561701	3900	3	1.46E-07	396	10	
DMR5:3879101	5	3879101	2700	2	6.99E-05	32	1.1	
DMR5:4311001	5	4311001	700	3	9.20E-06	19	2.7	
DMR5:9122501	5	9122501	700	2	1.21E-05	27	3.8	SEMA5A
DMR5:23303701	5	23303701	1700	2	7.07E-05	44	2.5	CTD-2272G21.2
DMR5:30068101	5	30068101	2900	2	1.28E-07	38	1.3	
DMR5:55530601	5	55530601	500	2	2.89E-05	28	5.6	PPAP2A;RNF138P1
DMR5:57549501	5	57549501	1400	2	1.42E-05	15	1	
DMR5:77912901	5	77912901	1300	2	3.39E-05	24	1.8	
DMR5:134314001	5	134314001	900	2	3.23E-05	10	1.1	CTD-2410N18.4;CDKL3
DMR5:151531101	5	151531101	1000	3	2.76E-06	44	4.4	FAT2
DMR5:162993101	5	162993101	800	2	1.10E-06	10	1.2	
DMR6:1514701	6	1514701	1100	2	1.70E-05	38	3.4	RP11-157J24.1
DMR6:5092901	6	5092901	300	2	4.79E-05	2	0.6	
DMR6:5792601	6	5792601	2400	2	1.74E-05	23	0.9	
DMR6:31326001	6	31326001	1000	2	3.38E-05	17	1.7	HLA-C
DMR6:31814701	6	31814701	3700	3	1.17E-05	217	5.8	HSPA1L;HSPA1A
DMR6:31828001	6	31828001	1600	2	2.62E-05	130	8.1	HSPA1B
DMR6:58560701	6	58560701	2100	2	4.49E-06	37	1.7	
DMR6:58593201	6	58593201	3200	2	6.04E-09	60	1.8	
DMR6:59033701	6	59033701	600	2	3.44E-11	13	2.1	
DMR6:59163101	6	59163101	1600	2	2.73E-06	34	2.1	
DMR6:59274801	6	59274801	3200	2	1.30E-07	57	1.7	
DMR6:59342901	6	59342901	1500	4	3.08E-06	36	2.4	
DMR6:59565501	6	59565501	1000	2	5.85E-06	12	1.2	
DMR6:59688201	6	59688201	200	2	7.98E-08	5	2.5	
DMR6:116616001	6	116616001	300	2	3.05E-06	10	3.3	
DMR6:167702601	6	167702601	2900	2	4.71E-05	70	2.4	
DMR7:49018601	7	49018601	700	3	9.21E-07	14	2	
DMR7:77441801	7	77441801	400	2	9.98E-07	6	1.5	
DMR7:87205701	7	87205701	2300	2	3.39E-05	37	1.6	TMEM243
DMR7:101239401	7	101239401	1600	2	6.96E-06	66	4.1	FIS1

DMR7:109351301	7	109351301	400	2	1.94E-05	9	2.2	
DMR7:158556901	7	158556901	1100	2	5.39E-06	65	5.9	PTPRN2
DMR8:7556101	8	7556101	500	2	8.30E-06	25	5	
DMR8:27797501	8	27797501	500	2	5.39E-05	3	0.6	ESCO2
DMR8:44360401	8	44360401	300	2	3.67E-05	6	2	
DMR8:45815901	8	45815901	300	2	9.64E-09	7	2.3	
DMR8:45927501	8	45927501	6800	2	3.41E-06	122	1.7	
DMR8:99694501	8	99694501	3100	3	5.63E-06	68	2.1	VPS13B;AC018442.1
DMR8:126213701	8	126213701	300	2	1.09E-05	2	0.6	
DMR8:142938801	8	142938801	1400	2	3.03E-05	55	3.9	
DMR9:28333101	9	28333101	1100	2	3.09E-06	8	0.7	LINGO2
DMR9:40951901	9	40951901	500	2	4.31E-05	16	3.2	
DMR9:41424201	9	41424201	600	2	7.72E-07	5	0.8	
DMR9:95044801	9	95044801	1100	2	5.16E-07	50	4.5	NPEPO
DMR9:98644001	9	98644001	1100	2	1.78E-05	12	1	GABBR2
DMR10:1197701	10	1197701	3100	8	1.51E-09	70	2.2	ADARB2
DMR10:15012201	10	15012201	1700	2	8.22E-05	42	2.4	
DMR10:30846501	10	30846501	2900	2	6.76E-05	40	1.3	ZNF438
DMR10:32731701	10	32731701	400	2	3.00E-05	14	3.5	CCDC7
DMR10:73117601	10	73117601	300	2	1.77E-05	1	0.3	NUDT13
DMR10:90990401	10	90990401	400	2	8.86E-05	4	1	
DMR10:123691901	10	123691901	800	2	1.57E-05	4	0.5	GPR26
DMR10:127538001	10	127538001	1800	2	2.69E-05	28	1.5	
DMR10:129343401	10	129343401	2100	3	6.38E-06	49	2.3	
DMR10:130440901	10	130440901	1100	2	2.10E-05	29	2.6	RP11-540N6.1
DMR11:484301	11	484301	4300	2	2.10E-06	312	7.2	PTDSS2
DMR11:47036401	11	47036401	1700	2	8.45E-06	33	1.9	C11orf49
DMR11:95437501	11	95437501	400	2	1.72E-05	8	2	
DMR12:81062701	12	81062701	400	2	4.28E-07	2	0.5	ACSS3
DMR12:95948901	12	95948901	300	2	1.85E-05	3	1	AMDHD1
DMR12:130657401	12	130657401	2600	2	1.48E-05	80	3	RP11-662M24.2
DMR13:63935201	13	63935201	300	2	8.12E-05	3	1	
DMR13:98815001	13	98815001	3000	2	6.61E-05	41	1.3	DOCK9
DMR13:104088001	13	104088001	500	2	2.27E-07	7	1.4	
DMR14:19433401	14	19433401	3600	3	2.54E-06	137	3.8	POTEG
DMR14:38737701	14	38737701	300	2	1.02E-06	5	1.6	
DMR14:46935601	14	46935601	300	2	5.76E-06	2	0.6	MDGA2
DMR14:62802301	14	62802301	500	2	1.46E-07	8	1.6	KCNH5
DMR15:20756701	15	20756701	2500	2	4.04E-05	46	1.8	
DMR15:21172601	15	21172601	3800	4	4.00E-07	119	3.1	
DMR15:21325001	15	21325001	5800	2	1.57E-06	135	2.3	RP11-32B5.7;RP11-275E15.2
DMR15:73775201	15	73775201	300	2	3.11E-06	6	2	
DMR15:88274701	15	88274701	300	2	7.18E-05	4	1.3	
DMR15:88930701	15	88930701	400	3	9.67E-09	1	0.2	
DMR16:2603101	16	2603101	1600	5	6.45E-07	77	4.8	AC141586.5;PDPK1
DMR16:14910901	16	14910901	2800	5	9.51E-06	217	7.7	MIR3180-1;NPIPA3
DMR16:61463701	16	61463701	1200	2	2.76E-06	2	0.1	
DMR16:85972901	16	85972901	1200	2	3.25E-05	95	7.9	
DMR17:121701	17	121701	6300	2	1.17E-05	115	1.8	
DMR17:8836901	17	8836901	2500	2	3.82E-06	53	2.1	PIK3R6
DMR17:46382701	17	46382701	1600	2	1.94E-05	14	0.8	NSFP1
DMR17:68151901	17	68151901	900	2	4.81E-05	30	3.3	LRRC37A16P
DMR18:8634901	18	8634901	200	2	1.21E-05	1	0.5	RAB12
DMR18:14484901	18	14484901	5100	2	1.18E-05	181	3.5	GRAMD4P7;CXADRP3
DMR18:46969701	18	46969701	200	2	2.87E-05	18	9	KATNAL2;TCEB3CL
DMR18:59752001	18	59752001	300	2	1.46E-06	3	1	
DMR18:65609101	18	65609101	600	2	2.08E-05	19	3.1	RP11-775G23.1
DMR18:70848101	18	70848101	900	2	1.62E-05	13	1.4	

DMR18:76003301	18	76003301	2800	2	2.92E-07	39	1.3	
DMR19:756801	19	756801	1600	2	3.50E-06	76	4.7	MISP
DMR19:37842601	19	37842601	2500	2	1.14E-05	36	1.4	AC016582.2
DMR19:43206901	19	43206901	400	2	2.88E-06	16	4	PSG4
DMR19:48181401	19	48181401	1600	2	7.54E-06	51	3.1	CARD8;ZNF114;C19
DMR19:52916701	19	52916701	4200	5	3.66E-10	101	2.4	ZNF888
DMR19:54772001	19	54772001	6000	4	8.20E-06	62	1	KIR2DL1;KIR3DL1: CTB
DMR20:2311001	20	2311001	800	2	7.56E-06	7	0.8	TGM3
DMR20:61964901	20	61964901	1500	2	8.95E-08	82	5.4	TAF4
DMR20:64131901	20	64131901	2700	3	1.68E-08	15	0.5	
DMR21:7916001	21	7916001	26500	5	1.05E-06	710	2.6	
DMR21:10652801	21	10652801	38000	3	9.10E-06	829	2.1	
DMR21:42955701	21	42955701	200	2	2.98E-05	12	6	
DMR21:44158201	21	44158201	1800	2	1.96E-05	51	2.8	AP001055.6
DMR22:11248401	22	11248401	8500	2	1.87E-05	383	4.5	5_8S_rRNA;AC137488.1
DMR22:32203601	22	32203601	3800	2	3.66E-06	159	4.1	RP1-90G24.10
DMR22:48701801	22	48701801	3100	2	3.52E-05	92	2.9	FAM19A5
DMRX:666401	X	666401	1000	2	1.66E-05	18	1.8	
DMRX:1041801	X	1041801	9200	4	1.75E-06	164	1.7	
DMRX:1235401	X	1235401	1100	2	1.72E-05	98	8.9	
DMRX:3865201	X	3865201	3100	2	8.41E-05	83	2.6	RP11-706O15.3
DMRX:115191101	X	115191101	1500	6	5.42E-07	128	8.5	LRCH2;RBMXL3
DMRY:11559901	Y	11559901	33000	3	9.16E-06	910	2.7	
