# Supplementary material for: Differential DNA Methylation Regions in Adult Human Sperm following Adolescent Chemotherapy: Potential for Epigenetic Inheritance
Source: PLoS One. 2017 Feb 1;12(2):e0170085. doi: 10.1371/journal.pone.0170085 (PMC5287489; doi:10.1371/journal.pone.0170085)
Supplement: S3 Table — The DMR name, gene symbol, chromosome location start and end position, Ensembl number, gene description and classification category are presented. (PDF) [file pone.0170085.s007.pdf]

Supplemental Table S3 Multiple Site DMR Associated Genes

DMR Name	Gene Symbol	Chr	start_position	end_position	Ensembl #	Gene Description	Classification Category
DMR1:12173801	TNFRSF1B	1	12167003	12209228	ENSG00000028137	tumor necrosis factor receptor superfamily - member 1B	Receptor
DMR1:175491101	TNR	1	175315194	175743770	ENSG00000116147	tenascin R	ECM
DMR1:215393901	RP11-199H2.2	1	215393646	215394418	ENSG00000282265	NA	Unknown
DMR1:224010101	RP11-504P24.3	1	223992743	224010612	ENSG00000185495	NA	Unknown
DMR2:2189101	MYT1L	2	1789113	2331260	ENSG00000186487	myelin transcription factor 1-like	Transcription
DMR2:144492201	ZEB2	2	144384081	144524583	ENSG00000169554	zinc finger E-box binding homeobox 2	Transcription
DMR2:238198201	ILKAP	2	238170401	238203729	ENSG00000132323	integrin-linked kinase-associated serine/threonine phosphatase	Signaling
DMR3:55470001	WNT5A	3	55465715	55490539	ENSG00000114251	wingless-type MMTV integration site family - member 5A	Development
DMR4:186476501	F11-AS1	4	186286094	186500997	ENSG00000251165	F11 antisense RNA 1	Epigenetic
DMR4:186476501	RP11-215A19.2	4	186426546	186555328	ENSG00000272297	Uncharacterized protein	Unknown
DMR4:188443001	LINC01060	4	188400736	188681051	ENSG00000249378	long intergenic non-protein coding RNA 1060	Epigenetic
DMR5:164501	PLEKHG4B	5	140258	189970	ENSG00000153404	pleckstrin homology domain containing - family G (with RhoGef domain) member 4B	Signaling
DMR5:9122501	SEMA5A	5	9035026	9546075	ENSG00000112902	sema domain - seven thrombospondin repeats (type 1 and type 1-like) - transmembrane domain (TM)	Signaling
DMR5:23303701	CTD-2272G21.2	5	23303565	23305143	ENSG00000250332	NA	Unknown
DMR5:55530601	PPAP2A	5	55424854	55535050	ENSG00000067113	phosphatidic acid phosphatase type 2A	Signaling
DMR5:55530601	RNF138P1	5	55530156	55530701	ENSG00000250853	ring finger protein 138 - E3 ubiquitin protein ligase pseudogene 1	Unknown
DMR5:134314001	CTD-2410N18.4	5	134205614	134371044	ENSG00000273345	NA	Unknown
DMR5:134314001	CDKL3	5	134286350	134371047	ENSG00000006837	cyclin-dependent kinase-like 3	Cell Cycle
DMR5:151531101	FAT2	5	151504093	151568944	ENSG00000086570	FAT atypical cadherin 2	ECM
DMR6:1514701	RP11-157J24.1	6	1513698	1515289	ENSG00000218027	NA	Unknown
DMR6:31326001	HLA-C	6	31268749	31357158	ENSG00000204525	major histocompatibility complex - class I - C	Immune
DMR6:31814701	HSPA1L	6	31809619	31815065	ENSG00000204390	heat shock 70kDa protein 1-like	Protein Binding
DMR6:31814701	HSPA1A	6	31815464	31817946	ENSG00000204389	heat shock 70kDa protein 1A	Protein Binding
DMR6:31828001	HSPA1B	6	31827735	31830255	ENSG00000204388	heat shock 70kDa protein 1B	Protein Binding
DMR7:87205701	TMEM243	7	87196160	87220587	ENSG00000135185	transmembrane protein 243 - mitochondrial	Mitochondria
DMR7:101239401	FIS1	7	101239458	101252316	ENSG00000214253	fission 1 (mitochondrial outer membrane) homolog (S. cerevisiae)	Mitochondria
DMR7:158556901	PTPRN2	7	157539056	158587788	ENSG00000155093	protein tyrosine phosphatase - receptor type - N polypeptide 2	Signaling
DMR8:27797501	ESCO2	8	27771949	27812640	ENSG00000171320	establishment of sister chromatid cohesion N-acetyltransferase 2	Epigenetic
DMR8:99694501	VPS13B	8	99013266	99877580	ENSG00000132549	vacuolar protein sorting 13 homolog B (yeast)	Signaling
DMR8:99694501	AC018442.1	8	99695957	99698017	ENSG00000235683	NA	Unknown
DMR9:28333101	LINGO2	9	27948078	28670286	ENSG00000174482	leucine rich repeat and Ig domain containing 2	ECM
DMR9:95044801	NPEPO	9	94726701	95087218	ENSG00000148120	chromosome 9 open reading frame 3	Proteolysis
DMR9:98644001	GABBR2	9	98288109	98709197	ENSG00000136928	gamma-aminobutyric acid (GABA) B receptor - 2	Receptor
DMR10:1197701	ADARB2	10	1177318	1737476	ENSG00000185736	adenosine deaminase - RNA-specific - B2 (non-functional)	Translation
DMR10:30846501	ZNF438	10	30820207	31031937	ENSG00000183621	zinc finger protein 438	Transcription
DMR10:32731701	CCDC7	10	32567723	32882874	ENSG00000150076	coiled-coil domain containing 7	Transcription
DMR10:73117601	NUDT13	10	73110375	73131828	ENSG00000166321	nudix (nucleoside diphosphate linked moiety X)-type motif 13	Metabolism
DMR10:12369190	GPR26	10	123666355	123694607	ENSG00000154478	G protein-coupled receptor 26	Receptor
DMR10:13044090	RP11-540N6.1	10	130439067	130483154	ENSG00000236303	NA	Unknown
DMR11:484301	PTDSS2	11	448268	491399	ENSG00000174915	phosphatidylserine synthase 2	Metabolism
DMR11:47036401	C11orf49	11	46936689	47164385	ENSG00000149179	chromosome 11 open reading frame 49	Unknown
DMR12:81062701	ACSS3	12	80936414	81261205	ENSG00000111058	acyl-CoA synthetase short-chain family member 3	Metabolism

DMR12:95948901	AMDHD1	12	95943293	95968716	ENSG00000139344	amidohydrolase domain containing 1	Metabolism
DMR12:13065740	RP11-662M24.2	12	130628316	130716281	ENSG00000256725	NA	Unknown
DMR12:13065740	RIMBP2	12	130396137	130716281	ENSG00000060709	RIMS binding protein 2	Misc.
DMR12:13065740	RP11-662M24.1	12	130651371	130669233	ENSG00000256343	NA	Unknown
DMR13:98815001	DOCK9	13	98793487	99086625	ENSG00000088387	dedicator of cytokinesis 9	Signaling
DMR14:19433401	POTEG	14	19402486	19434341	ENSG00000187537	POTE ankyrin domain family - member G	Unknown
DMR14:46935601	MDGA2	14	46839629	47674954	ENSG00000139915	MAM domain containing glycosylphosphatidylinositol anchor 2	Metabolism
DMR14:62802301	KCNH5	14	62699454	63102037	ENSG00000140015	potassium channel - voltage gated eag related subfamily H - member 5	Transport
DMR15:21325001	RP11-32B5.7	15	21298233	21325241	ENSG00000247765	NA	Unknown
DMR15:21325001	RP11-275E15.2	15	21328380	21343881	ENSG00000280881	NA	Unknown
DMR16:2603101	AC141586.5	16	2603350	2630494	ENSG00000215154	NA	Unknown
DMR16:2603101	PDPK1	16	2537964	2603188	ENSG00000140992	3-phosphoinositide dependent protein kinase 1	Signaling
DMR16:14910901	MIR3180-1	16	14911220	14911313	ENSG00000265537	microRNA 3180-1	Epigenetic
DMR16:14910901	NPIPA3	16	14708944	14952073	ENSG00000224712	nuclear pore complex interacting protein family - member A3	Unknown
DMR16:14910901	RP11-958N24.1	16	14911551	14935708	ENSG00000183458	NA	Unknown
DMR16:14910901	NPIPA1	16	14750813	14952060	ENSG00000183426	nuclear pore complex interacting protein family - member A1	Unknown
DMR17:8836901	PIK3R6	17	8802723	8867677	ENSG00000276231	phosphoinositide-3-kinase - regulatory subunit 6	Signaling
DMR17:46382701	NSFP1	17	46372855	46487141	ENSG00000260075	N-ethylmaleimide-sensitive factor pseudogene 1	Unknown
DMR17:68151901	LRRC37A16P	17	68125777	68152468	ENSG00000267023	leucine rich repeat containing 37 - member A16 - pseudogene	Unknown
DMR17:68151901	RP11-147L13.7	17	68152776	68159043	ENSG00000267708	NA	Unknown
DMR18:8634901	RAB12	18	8609445	8639381	ENSG00000206418	RAB12 - member RAS oncogene family	Signaling
DMR18:14484901	GRAMD4P7	18	14485806	14487501	ENSG00000266242	GRAM domain containing 4 pseudogene 7	Unknown
DMR18:14484901	CXADRP3	18	14477955	14499278	ENSG00000265766	coxsackie virus and adenovirus receptor pseudogene 3	Unknown
DMR18:46969701	KATNAL2	18	46917492	47102243	ENSG00000167216	katanin p60 subunit A-like 2	Cytoskeleton
DMR18:46969701	TCEB3CL	18	46968695	47029842	ENSG00000275553	transcription elongation factor B polypeptide 3C-like	Transcription
DMR18:65609101	RP11-775G23.1	18	65606090	65652053	ENSG00000265217	NA	Unknown
DMR19:756801	MISP	19	751126	764318	ENSG00000099812	mitotic spindle positioning	Cell Cycle
DMR19:37842601	AC016582.2	19	37823722	37855215	ENSG00000225868	NA	Unknown
DMR19:43206901	PSG4	19	43192702	43207299	ENSG00000243137	pregnancy specific beta-1-glycoprotein 4	ECM
DMR19:48181401	CARD8	19	48180770	48255946	ENSG00000105483	caspase recruitment domain family - member 8	Apoptosis
DMR19:48181401	ZNF114	19	48172318	48287608	ENSG00000178150	zinc finger protein 114	Transcription
DMR19:48181401	C19orf68	19	48170692	48197620	ENSG00000185453	chromosome 19 open reading frame 68	Unknown
DMR19:52916701	ZNF888	19	52915196	52923470	ENSG00000213793	zinc finger protein 888	Transcription
DMR19:54772001	KIR2DL1	19	54769811	54784322	ENSG00000125498	killer cell immunoglobulin-like receptor - two domains - long cytoplasmic tail - 1	Immune
DMR19:54772001	KIR3DL1	19	54724497	54867215	ENSG00000167633	killer cell immunoglobulin-like receptor - three domains - long cytoplasmic tail - 1	Immune
DMR19:54772001	CTB-61M7.1	19	54724496	54798285	ENSG00000215765		Unknown
DMR20:2311001	TGM3	20	2296001	2341078	ENSG00000125780	transglutaminase 3 [Source:HGNC Symbol;Acc:HGNC:11779]	Metabolism
DMR20:61964901	TAF4	20	61953469	62065810	ENSG00000130699	TAF4 RNA polymerase II - TATA box binding protein (TBP)-associated factor - 135kDa	Transcription
DMR21:44158201	AP001055.6	21	44158740	44160076	ENSG00000225331	NA	Unknown
DMR22:11248401	5_8S_rRNA	22	11249809	11249959	ENSG00000276871	5.8S ribosomal RNA [Source:RFAM;Acc:RF00002]	Translation
DMR22:11248401	AC137488.1	22	11253605	11253719	ENSG00000277683		Unknown
DMR22:32203601	RP1-90G24.10	22	32205115	32269666	ENSG00000242082	NA	Unknown
DMR22:48701801	FAM19A5	22	48489460	48850912	ENSG00000219438	family with sequence similarity 19 (chemokine (C-C motif)-like) - member A5	Growth Factor
DMRX:3865201	RP11-706O15.3	X	3853010	3882317	ENSG00000234449	NA	Unknown
DMRX:115191101	LRCH2	X	115110616	115234072	ENSG00000130224	leucine-rich repeats and calponin homology (CH) domain containing 2	Cytoskeleton
DMRX:115191101	RBMXL3	X	115189427	115192868	ENSG00000175718	RNA binding motif protein - X-linked-like 3	Translation
